# Right ventricular dyssynchrony in patients with pulmonary hypertension is associated with disease severity and functional class

**DOI:** 10.1186/1476-7120-3-23

**Published:** 2005-08-29

**Authors:** Angel López-Candales, Kaoru Dohi, Navin Rajagopalan, Matthew Suffoletto, Srinivas Murali, John Gorcsan, Kathy Edelman

**Affiliations:** 1 Cardiovascular Institute at the University of Pittsburgh Medical Center, Pittsburgh, PA, USA

**Keywords:** Dyssynchrony, right ventricle, outcomes, pulmonary hypertension, strain imaging, tissue Doppler imaging

## Abstract

**Background:**

Abnormalities in right ventricular function are known to occur in patients with pulmonary arterial hypertension.

**Objective:**

Test the hypothesis that chronic elevation in pulmonary artery systolic pressure delays mechanical activation of the right ventricle, termed dyssynchrony, and is associated with both symptoms and right ventricular dysfunction.

**Methods:**

Fifty-two patients (mean age 46 ± 15 years, 24 patients with chronic pulmonary hypertension) were prospectively evaluated using several echocardiographic parameters to assess right ventricular size and function. In addition, tissue Doppler imaging was also obtained to assess longitudinal strain of the right ventricular wall, interventricular septum, and lateral wall of the left ventricle and examined with regards to right ventricular size and function as well as clinical variables.

**Results:**

In this study, patients with chronic pulmonary hypertension had statistically different right ventricular fractional area change (35 ± 13 percent), right ventricular end-systolic area (21 ± 10 cm^2^), right ventricular Myocardial Performance Index (0.72 ± 0.34), and Eccentricity Index (1.34 ± 0.37) than individuals without pulmonary hypertension (51 ± 5 percent, 9 ± 2 cm^2^, 0.27 ± 0.09, and 0.97 ± 0.06, p < 0.005, respectively). Furthermore, peak longitudinal right ventricular wall strain in chronic pulmonary hypertension was also different -20.8 ± 9.0 percent versus -28.0 ± 4.1 percent, p < 0.01). Right ventricular dyssynchrony correlated very well with right ventricular end-systolic area (r = 0.79, p < 0.001) and Eccentricity Index (r = 0.83, p < 0.001). Furthermore, right ventricular dyssynchrony correlates with pulmonary hypertension severity index (p < 0.0001), World Health Organization class (p < 0.0001), and number of hospitalizations (p < 0.0001).

**Conclusion:**

Lower peak longitudinal right ventricular wall strain and significantly delayed time-to-peak strain values, consistent with right ventricular dyssynchrony, were found in a small heterogeneous group of patients with chronic pulmonary hypertension when compared to individuals without pulmonary hypertension. Furthermore, right ventricular dyssynchrony was associated with disease severity and compromised functional class.

## Background

Right ventricular systolic dysfunction has been identified as a key element in determining prognosis of patients afflicted with chronic pulmonary hypertension [1-7]. Although echocardiography has proved invaluable to noninvasively assess pulmonary artery pressures; evaluation of right ventricular size and systolic function by echocardiography is somewhat more difficult, largely because of the complex RV anatomy that limits its evaluation [8-14]. Therefore, identification of early right ventricular dysfunction is of outmost clinical importance since as many as two-thirds of the deaths in patients with chronic pulmonary hypertension may be attributed to right ventricular failure [15-19].

The recent introduction of strain and strain rate echocardiography using tissue Doppler imaging (TDI) has provided an objective means to quantify global and regional left ventricular function with improved accuracy and greater reproducibility than conventional echocardiography [20-23]. We have recently reported that TDI is also useful in identifying right ventricular free wall mechanical delay in patients with chronic pulmonary hypertension [24]. However, its clinical significance and potential relevance remains to be determined. We therefore designed this study to answer two critical questions. First, determine if right ventricular dyssynchrony in patients with chronic pulmonary hypertension is associated with indices of disease severity and impaired functional class when compared to individuals without pulmonary hypertension. Second, determine if TDI can identify right ventricular dyssynchrony in patients with chronic pulmonary hypertension before any visible abnormalities of right ventricular size or function are apparent by using routine transthoracic echocardiography.

## Methods

### Study population

Fifty-two patients (mean age 46 ± 15 years, 22 males) who were referred to our echocardiographic laboratory underwent a complete echocardiographic examination. In the population studied, 24 patients had chronic pulmonary hypertension, as determined by echocardiography [25], including 8 patients with parenchymal lung disease, 4 patients with idiopathic pulmonary hypertension, 5 patients with chronic thrombo-embolic pulmonary hypertension, 3 patients with porto-pulmonary hypertension, and 4 patients with connective tissue disorder. In these patients with chronic pulmonary hypertension, all electronic hospital records were retrospectively reviewed to assess how often these patients were hospitalized, seen in the emergency room visits, or had evidence of clinical deterioration for a period of 6-months prior to the echocardiogram.

Patients with an irregular heart rhythm such as atrial fibrillation, history of significant coronary artery disease, previous myocardial infarction, resting wall motion abnormalities, cardiomyopathy, abnormal left ventricular systolic function, valvular heart disease or the presence of a pacer or defibrillator wire in the right ventricle were all excluded.

The Institutional Review Board of the University of Pittsburgh Medical Center approved the study and all patients gave informed consent.

### Standard echocardiography

All patients underwent a complete transthoracic echocardiographic study including two-dimensional, color flow and spectral Doppler as well as tissue strain imaging using a GE-Vingmed Vivid 7 system (GE Vingmed Ultrasound, Horten, Norway). Standard two-dimensional echocardiographic evaluation of RV size and function was performed as routinely [26]. In addition, right ventricular end-diastolic and end-systolic areas were measured from the apical 4-chamber view to calculate right ventricular fractional area change. Eccentricity Index using the mid-ventricular short axis image at the level of the papillary muscles in both systole and diastole and right ventricular Myocardial Performance Index were calculated as previously reported [19,26,27].

Pulmonary artery systolic pressures were estimated using the approach of calculating the systolic pressure gradient between right ventricle and right atrium by the maximum velocity of the tricuspid regurgitant jet using the modified Bernoulli equation and then adding to this value an estimated right atrial pressures based on both the size of the inferior vena cava and the change in caliber of this vessel with respiration [25,28].

To verify that the severity of tricuspid regurgitation was reliable, we also determined the vena contracta width as previously documented by Tribouilloy et al [29]; specifically the position of the transducer was modified to optimize visualization of the flow convergence region and the regurgitant flow proximal and distal to the tricuspid valve, the aliasing velocity ranged from 46 to 96 cm/s and the narrowest neck of the regurgitant flow just distal to the flow convergence region was measured in mid systole by an observer unaware of the clinical examination.

### Tissue Doppler

Color-coded tissue Doppler cine loops were obtained as routinely performed in our laboratory from 3 beats obtained from apical 4-chamber views at the depths of 14 ± 2 cm with pulse repetition frequency set at 1 kHz, Nyquist velocity range ± 16 cm/sec and frame rates 99 ± 9 Hz [20,30,31]. Initial length for longitudinal strain measurement was set at 12 mm and the regions of interest with a length of 20 to 24 mm and width of 6 to 8 mm were placed in the basal and mid segments of the right ventricular lateral free wall (RVw), inter-ventricular septum (IS), and left ventricular lateral (LVL) wall to measure the peak longitudinal systolic strain and time-to-peak strain from the onset of Q-wave on the electrocardiogram and shown as mean values for both basal and mid segments for the corresponding right and left ventricular walls. Right and left ventricular dyssynchrony was determined as the difference in time-to-peak strain from IS to RVw and from IS to LVL. We also determine difference in right to left ventricular synchrony as the differences in time-to-peak strain from RVF to LVL. Finally, longitudinal right ventricular annular displacement was also measured by placing transducer region of interest with a 7 by 7 mm sample volume in the junction of the right ventricular free wall and the tricuspid valve.

### Statistical analysis

All echocardiographic parameters were calculated using the commercially available software EchoPAC PC version 3.00 (GE Vingmed Ultrasound) and determined by a single observer. All intervals were corrected for heart rate (corrected interval = measured interval/ (RRinterval)^1/2^) [30,31]. Group data (mean ± SD) were compared using the 2-tailed Student's *t*-test for paired and unpaired data, respectively. Linear regression analysis was used to examine relations between various dependent variables. Univariate analysis of right ventricular dyssynchrony to clinical and echocardiographic variables was also performed. To assess if there was any correlation between right ventricular dyssynchrony and clinical variables, we gathered information regarding World Health Organization (WHO) symptom class, systolic pulmonary artery pressure (SPAP) severity index, and hospitalizations due to pulmonary hypertension or heart failure symptoms over a 12-month period prior to the echocardiographic examination. We determined SPAP severity index as follows: A pulmonary hypertension severity of 1 corresponded to a SPAP 36–50; 2 as a SPAP 51–75; and 3 as a SPAP > 75. P-values of less than .05 were considered to be statistically significant.

## Results

Echocardiographic results obtained in all 52 patients are summarized in Table 1. Although the mean values for all standard echocardiographic parameters to assess right ventricular size and function were found within normal limits; there was a wide spread of values. In the case of right ventricular end-diastolic areas the mean value obtained for the entire population studied was 26 ± 10 cm^2^, with a minimum value of 12 and a maximum value of 59 cm^2^. Similarly, the mean value for right ventricular end-systolic areas was 16 ± 10 cm^2 ^but ranged from 5 to 45 cm^2^; consequently the calculated mean right ventricular fractional area change was also found within normal (43 ± 13), with a minimum value of 13 and a maximum value of 65. With regards to Eccentricity Index, the mean value was 1.16 ± 0.32 and included values from 0.84 to 3.28. Similarly, the Myocardial Performance Index was 0.53 ± 0.35 but ranged from 0.13 to 1.26. For the whole population studied, the mean pulmonary artery systolic pressure was 55 ± 33 mmHg, ranging from 15 to 116 mmHg. Since most healthy subjects only have a trivial amount of tricuspid regurgitation, estimation of peak pulmonary artery systolic pressures, in these individuals was not possible. However, the mean vena contracta width in patients with chronic pulmonary hypertension was 5.7 ± 2.2 mm.

**Table 1:** Standard two-dimensional echocardiographic and Doppler data.

**Parameters**	**Population Studied**
Standard Echocardiography	
RV end diastolic area (cm^2^)	26 ± 10
RV end systolic area (cm^2^)	16 ± 10
RV fractional area change (%)	43 ± 13
Eccentricity index	1.16 ± 0.32
Myocardial performance index	0.53 ± 0.35
Pulmonary artery systolic pressure (mmHg)	53 ± 33
Left ventricular systolic function (%)	57 ± 5

**Figure 1:**
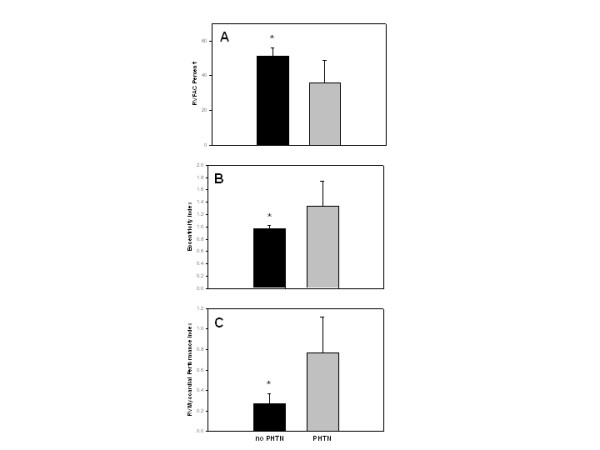
(A) Right ventricular fractional area change, expressed as a percentage, is significantly higher in individuals with no pulmonary hypertension (no PHTN), as demonstrated by the black columns, than in patients with chronic pulmonary hypertension (PHTN), as seen in the grey columns. (B) A significantly lower Eccentricity Index, as expected, is noted in individuals with no pulmonary hypertension when compared to patients with chronic pulmonary hypertension. (C) Similarly, a lower right ventricular Myocardial Performance Index is noted among normal individuals when compared to patients with chronic pulmonary hypertension.

It is only when we analyze this echocardiographic data according to the presence or absence of chronic pulmonary hypertension that certain correlations become apparent. As seen in Figure 1A, right ventricular fractional area change is significantly higher in individuals without pulmonary hypertension when compared to patients with chronic pulmonary hypertension. A significantly lower Eccentricity is noted among individuals without pulmonary hypertension when compared to patients with chronic pulmonary hypertension as seen in Figure 1B. Similarly, a lower RV Myocardial Performance Index (Figure 1C) is also noted among normal individuals when compared to patients with chronic pulmonary hypertension.

**Figure 2:**
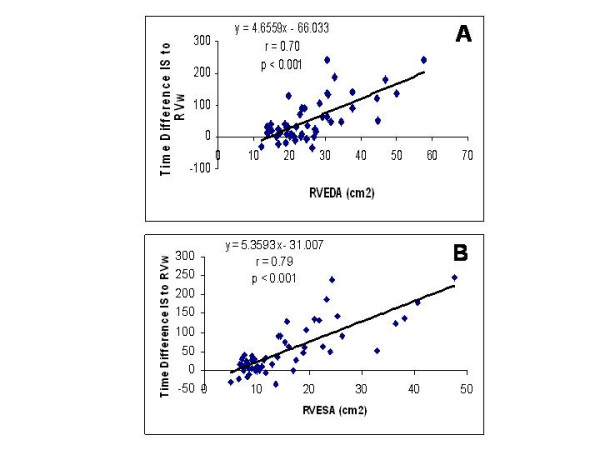
(A) A very strong correlation is shown between time differences between interventricular septum and the RVw, measured in ms, against the right ventricular end-diastolic area (R = 0.70, p < 0.001). (B) This graph shows the correlation between the time difference between interventricular septum and RVw activation and right ventricular end-systolic area (R = 0.79, p < 0.001).

**Figure 3:**
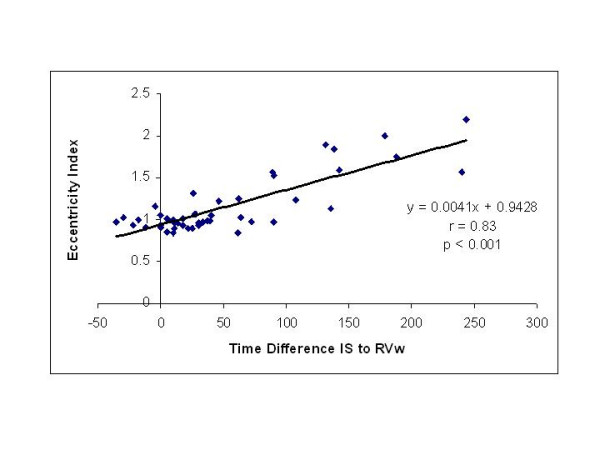
A very strong correlation is seen between Eccentricity Index (EI) and the time difference between interventricular septum and the RVw (R = 0.83, p < 0.001).

**Figure 4:**
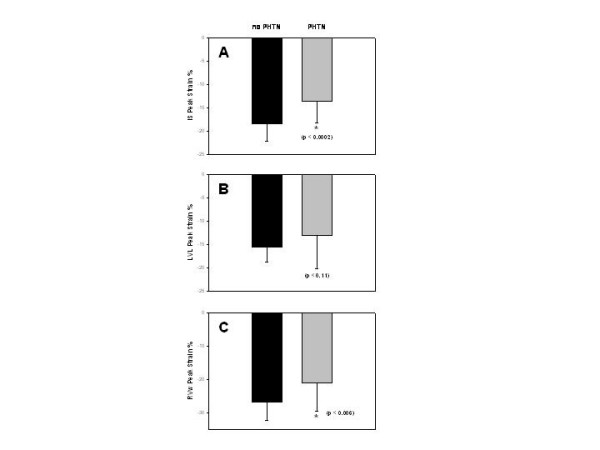
(A) Bar graph showing the mean and standard deviation values for individuals without pulmonary hypertension (black bars) and for patients with chronic pulmonary hypertension (grey bars) with regards to peak strain measured by TDI expressed in percent for the interventricular septum (IS). (B) Peak strain for the left ventricular lateral (LVL) wall and (C) and peak strain for the right ventricular wall (RVw). The p value for each one is represented.

**Figure 5:**
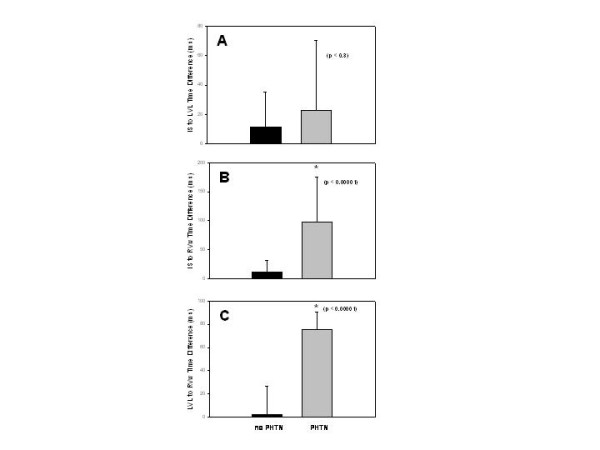
(A) Bar graph showing the mean and standard deviation values for individuals without pulmonary hypertension (black bars) and for patients with chronic pulmonary hypertension (grey bars) with regards to time difference in mechanical activation expressed in milliseconds (ms) between the interventricular septum (IS) and left ventricular lateral (LVL). (B) Time difference in mechanical activation between interventricular septum (IS) and right ventricular wall (RVw). (C) Time difference in mechanical activation between the left ventricular lateral (LVL) wall and the right ventricular wall (RVw). The p value for each one is represented.

A complete TDI examination was also obtained in all 52 patients and the results are summarized in Table 2. To better understand the echocardiographic findings based on TDI data we compared time differences between interventricular septum and the RVw, expressed in milliseconds (ms), against the right ventricular end-diastolic area and noted a very strong correlation (r = 0.70, p < 0.001) as shown in Figure 2A. An even better correlation was noted in the time difference between interventricular septum and the RVw activation when compared to right ventricular end-systolic area (r = 0.79, p < 0.001) as shown in Figure 2B. On Figure 3, a very strong correlation is noted between Eccentricity Index and the time difference between interventricular septum and the RVw (r = 0.83, p < 0.001). Finally, a better representation of the statistically significant differences between individuals without pulmonary hypertension and patients with chronic pulmonary hypertension is seen in both Figure 4 and 5. In Figure 4, we demonstrate the differences in peak strain, expressed in percent, for the RVw, IS, and LVL. It is important to note the statistically significant difference in peak strain generation between individuals without pulmonary hypertension and patients with chronic pulmonary hypertension with regards to both IS (Figure 4A) and RVw Figure 4C). In Figure 5, we demonstrate the time differences in mechanical activation, expressed in ms, between IS and LVL, IS and RVw, and LVL and RVw. A statistically significant difference in time difference of mechanical activation between individuals without pulmonary hypertension and patients with chronic pulmonary hypertension was only seen for both IS and RVw (Figure 5B) and LVL and RVw (Figure 5C), suggestive of the presence of right ventricular dyssynchrony. It is also demonstrated in Figure 5A, the presence of normal synchrony in the left ventricle.

**Table 2:** Tissue Doppler imaging data.

**Parameters**	**Population Studied**
Tissue Doppler Imaging	
Peak strain (%)	
RVw	-24 ± 8
IS	-16 ± 5
LVL	-14 ± 5
Time to peak strain (ms)	
RVw	417 ± 62
IS	364 ± 39
LVL	380 ± 47
Time difference (ms)	
IS – RVw	53 ± 66
IS – LVL	16 ± 37
RVw – LVL	37 ± 65

**Figure 6:**
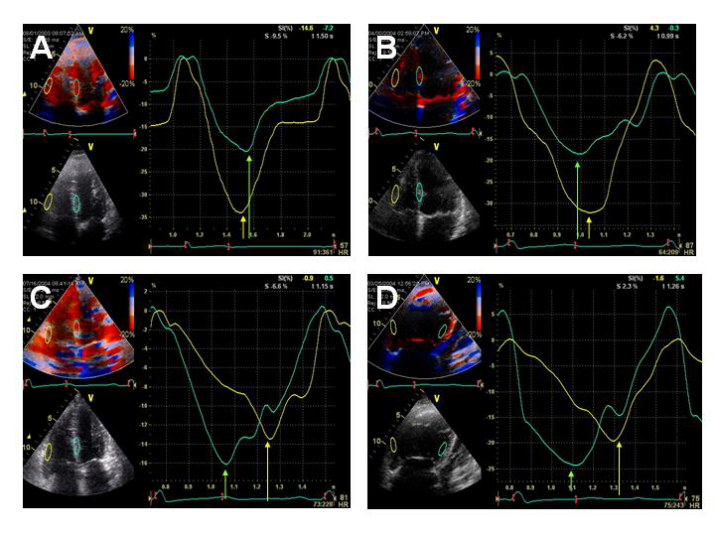
A representative tissue Doppler image is shown demonstrating (A) Almost synchronized time-to-peak longitudinal peak strain of both right ventricular free wall (yellow curve) and ventricular septum (green curve) in an individual without PAH. (B) Slightly delayed time-to-peak longitudinal right ventricular free wall peak strain (yellow curve) when compared to ventricular septum peak strain (green curve) in a patient with mild PAH. Note that there is no significant reduction in RVw strain generation. (C) A more noticeable delayed time-to-peak longitudinal right ventricular free wall peak strain (yellow curve) when compared to ventricular septum peak strain (green curve) is now evident in a patient with moderate PAH as well as in (D) in a patient with severe PAH and severe RV dysfunction. Note that in both (C) and (D) there is a significant reduction in RVw strain generation.

A representative tissue Doppler image is shown in Figure 6 demonstrating (A) Almost synchronized time-to-peak longitudinal peak systolic strain generation of both right ventricular free wall (yellow curve) and ventricular septum (green curve) in an individual without pulmonary hypertension. (B) A slightly delayed time-to-peak longitudinal right ventricular free wall peak systolic strain generation (yellow curve) is already noticeable when compared to the ventricular septum peak systolic strain (green curve) generation in a patient with mild chronic pulmonary hypertension. Please note that in both (A) and (B) the peak systolic strain (yellow curve) generated by the right ventricular wall when is higher than the peak systolic strain generated by the inter-ventricular septum. (C) A more noticeable delayed time-to-peak longitudinal right ventricular free wall peak systolic strain (yellow curve) generation when compared to ventricular septum peak systolic strain (green curve) generation is now evident in a patient with moderate chronic pulmonary hypertension as well as in (D) in a patient with severe chronic pulmonary hypertension and severe right ventricular dysfunction. In both (C) and (D), we can appreciate a significant reduction in peak systolic strain (yellow curve) generated by the right ventricular wall when compared to the peak systolic strain generated by the inter-ventricular septum (IS) as seen in the green curve in these patients with moderately severe pulmonary hypertension.

It is important to note that the electrocardiographic QRS duration between patients with chronic pulmonary hypertension was no different than the electrocardiographic QRS duration of individuals without pulmonary hypertension (91 ± 13 vs. 86 ± 9 ms, p = NS).

Univariate analysis between right ventricular dyssynchrony and extent of disease severity markers including World Health Organization (WHO) functional class, systolic pulmonary artery pressure (SPAP), hospitalizations, and deaths between individuals without pulmonary hypertension and patients with chronic pulmonary hypertension is seen in Table 3. Despite being a small sample we found that right ventricular dyssynchrony correlated significantly with WHO class, with SPAP severity index, and with the number of hospitalizations. Although there was also a correlation seen in terms of the number of deaths among patients with chronic pulmonary hypertension, the number of deaths is too small to be confident.

**Table 3:** shows data analysis regarding right ventricular dyssynchrony and extent of disease severity markers including World Health Organization (WHO) functional class, systolic pulmonary artery pressure (SPAP), hospitalizations, and deaths in individuals without pulmonary hypertension and patients with chronic pulmonary hypertension.

**Variables**	**No Pulmonary Hypertension**	**Chronic Pulmonary Hypertension**	**P value**
WHO class	1 ± 0	2.9 ± 0.9	0.0001
SPAP severity	0	2.5 ± 0.8	0.0001
Hospitalizations	0	33	0.0001
Deaths	0	3	0.005

## Discussion

The results of this study suggest that right ventricular dyssynchrony, represented as the time difference from interventricular septal to RVw activation [24], occurs in patients with chronic pulmonary hypertension and is strongly correlated with markers of right ventricular size and Eccentricity Index, a well-known parameter of right ventricular pressure overload [14]. In addition, the presence of right ventricular dyssynchrony correlates with markers disease severity including pulmonary hypertension severity index, World Health Organization class, and number of hospitalizations. Finally, right ventricular dyssynchrony is clearly evident with mild elevations in the pulmonary artery systolic pressure even when standard echocardiographic indices of right ventricular size and function are still within normal limits.

It is important to emphasize that in this study, right ventricular dyssynchrony was present even with a normal electrocardiographic QRS interval duration. A finding that is in agreement with previous data stating that an abnormal electrical conduction is not necessarily needed to produce left ventricular mechanical dyssynchrony; since left ventricular dyssynchrony has been identified in the failing myocardium with a normal QRS duration [32-34]. Therefore, it appears that not all contributing mechanisms resulting in mechanical dyssynchrony have been identified and are probably complex.

The results of this study can be quite useful in the evaluation of chronic pulmonary hypertension patients given the well-know limitations of standard echocardiography in the assessment of right ventricular size and function due to the complex structure and asymmetrical shape of this cardiac chamber [8-14]. We used changes in right ventricular area obtained from the apical 4-chamber views as indices of right ventricular size and global systolic function rather than ventricular volumes and ejection fraction. In addition we also used Myocardial Performance Index, a well-recognized measure of global systolic and diastolic function that is independent on any geometric assumptions and heart rate [26,27]. Finally, the use of the Eccentricity Index that indicates the degree of ventricular septal displacement is also a well-recognized marker of right ventricular deformation by either pressure or volume loads [14]. The variability in our measurements of RV size, morphology, and functional performance in patients with variable degree of chronic pulmonary hypertension is probably due to the right ventricular geometric remodeling that occurs in these patients with pulmonary hypertension as recently described by Sukmawan and associates [35].

The value of TDI has been widely applied to quantify regional left ventricular myocardial function under different clinical scenarios and all the available evidence suggests that it is quite useful to assess left ventricular mechanical dyssynchrony [36,37]. However, despite all this knowledge on left ventricular mechanical activation, no attempts have been made to quantify right ventricular dyssynchrony using strain imaging. In our study, we evaluated longitudinal shortening and its time sequence using strain imaging from apical 4-chamber views for two reasons; ease of accessibility from this window and functional dominancy of the longitudinal shortening over short-axis shortening [38].

Potential limitations of this present study obviously include the small number of patients. However, even with this small number of patients we were able to reach our primary goal of identifying the presence of a statistically significant right ventricular mechanical dyssynchrony in patients with chronic pulmonary hypertension. Second, the presence of a heterogeneous population of patients with regards to the etiology of their pulmonary hypertension. At first hand, this might be a crucial disadvantage; since the time sequence of events might be different with regards to different etiologies; however, what appears evident is that regardless of the initiating mechanism the same final pathway abnormality in right ventricular mechanics might be not that dissimilar between different etiologies. Third, an invasive pressure measurement was not used in this study; therefore, assessments of right ventricular time-pressure plots, dp/dt, and pulmonary vascular resistance were not available to compare with our echo and TDI data. Similarly, peak systolic pulmonary arterial pressures were estimated simply based on tricuspid regurgitation measurements. However, this widely used Doppler-derived pressure estimation is well recognized and has been documented to have a good correlation with simultaneously obtained catheter-derived measurements; particularly in patients with elevated systolic pulmonary artery pressures [39]. Last, the use of fractional area change as an index of global right ventricular systolic function has a limitation of being highly afterload dependent particularly in patients with pulmonary hypertension [40,41]. This effect might be compounded by tricuspid regurgitation that by reducing systolic afterload augments right ventricular systolic function. However, in this study we also assessed the severity of tricuspid regurgitation by measuring the width of the vena contracta and found the width of the vena contracta correlated with decreasing right ventricular fractional area change rather than playing role in augmenting right ventricular function.

It is important to clarify that right ventricular dyssynchrony was due to delayed RVw peak strain rather than to a septal motion abnormality. In addition, we found no significant dyssynchrony between septal to LV lateral activation in these patients. Although a clear mechanism to explain the delayed RVw contractility is beyond the scope of this study, we speculate that either ischemia with consequent tethering (post-systolic shortening) of the RVw or differences in afterload-dependency of the right ventricular free wall when compared to the interventricular septum might be considered possible mechanisms. In fact, post-systolic shortening of the RVw was observed in 50% of patients with chronic pulmonary hypertension in this study.

## Conclusion

We conclude that right ventricular dyssynchrony, represented as the time difference from interventricular septal to RVw activation, occurs in patients with chronic pulmonary hypertension and is strongly correlated with markers of right ventricular size and Eccentricity Index. In addition, right ventricular dyssynchrony is associated with disease severity as it correlates with pulmonary hypertension severity index, World Health Organization class, and number of hospitalizations. Finally, right ventricular dyssynchrony is clearly evident with mild elevations in the pulmonary artery systolic pressure even when standard echocardiographic indices of right ventricular size and function are still within normal limits. Therefore, it is tempting to suggest that TDI might be useful in the early identification of patients with subclinical evidence of right ventricular dysfunction but further studies are required. In addition, the long-term effects of right ventricular dyssynchrony on morbidity and mortality as well as whether right ventricular resynchronization therapy that might correct right ventricular dyssynchrony and restore right ventricular function with resultant improvement of markers of disease severity and functional capacity also require investigation.

## List of Abbreviations

TDI = Tissue Doppler imaging

RVw = Right ventricular lateral free wall

IS = Inter-ventricular septum

LVL = Left ventricular lateral wall

WHO = World Health Organization

SPAP = Systolic pulmonary artery pressure

dp/dt = Delta pressure / delta time

ms = milliseconds

## Competing interests

The author(s) declare that they have no competing interests.

## Authors' contributions

All the listed authors contributed to gather and interpret data as well as to write the full length of this manuscript.
